# RUBubbles as a novel tool to study categorization learning

**DOI:** 10.3758/s13428-021-01695-2

**Published:** 2021-10-20

**Authors:** Aylin Apostel, Jonas Rose

**Affiliations:** Department of Psychology, Neural Basis of Learning, Ruhr University Bochum, Universitaetsstrasse 150, GA 04/146, 44801 Bochum, Germany

**Keywords:** Categorization learning, Prototype- vs. exemplar-based training approach, MATLAB, Automated stimulus generation, (Visual) similarity, Continuous categories, Category exceptions, Various abstraction levels, Artificial category, Method, Custom code, GUI/app, Toolbox

## Abstract

Grouping objects into discrete categories affects how we perceive the world and represents a crucial element of cognition. Categorization is a widespread phenomenon that has been thoroughly studied. However, investigating categorization learning poses several requirements on the stimulus set in order to control which stimulus feature is used and to prevent mere stimulus–response associations or rote learning. Previous studies have used a wide variety of both naturalistic and artificial categories, the latter having several advantages such as better control and more direct manipulation of stimulus features. We developed a novel stimulus type to study categorization learning, which allows a high degree of customization at low computational costs and can thus be used to generate large stimulus sets very quickly. ‘RUBubbles’ are designed as visual artificial category stimuli that consist of an arbitrary number of colored spheres arranged in 3D space. They are generated using custom MATLAB code in which several stimulus parameters can be adjusted and controlled separately, such as number of spheres, position in 3D-space, sphere size, and color. Various algorithms for RUBubble generation can be combined with distinct behavioral training protocols to investigate different characteristics and strategies of categorization learning, such as prototype- vs. exemplar-based learning, different abstraction levels, or the categorization of a sensory continuum and category exceptions. All necessary MATLAB code is freely available as open-source code and can be customized or expanded depending on individual needs. RUBubble stimuli can be controlled purely programmatically or via a graphical user interface without MATLAB license or programming experience.

**Graphical abstract:**

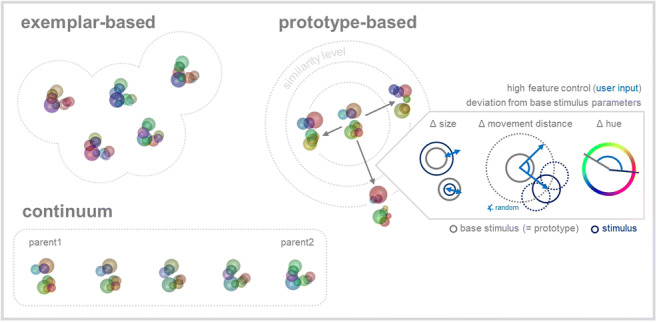

## Introduction

Being able to observe the weather through your kitchen window and to select an appropriate outfit saves yourself the trouble of running back home after a few minutes to fetch your umbrella. However, when leaving your favorite café after your morning coffee you might confuse your own umbrella with one of the others in the stand next to the door. We are constantly surrounded by an endless number of different contexts and objects that we automatically group into distinct categories. Whereas some categories seem quite abstract or vague, such as ‘good’ or ‘bad’ weather, others include very similar-looking, distinct objects such as ‘umbrella’.

Categorization describes the ability to group sensory stimuli into meaningful categories based on shared characteristics (Freedman & Miller, 2008; Jitsumori & Delius, 2001; Wutz et al., 2018). By definition, distinct objects that belong to the same class or category are treated equivalently and differently from objects in a different category (Mervis & Rosch, 1981). This behavior is generalized to novel objects of either category (Herrnstein, 1990; Jitsumori & Delius, 2001). Focusing on central features that are common in different objects within one category and ignoring irrelevant details reduces memory load and simultaneously influences how objects are perceived (DeGutis & D’Esposito, 2007; Goldstone, 1994). You might still remember that you brought an umbrella with you, however, without remembering whether it was dark blue or black.

Perceptual categorization is not restricted to humans, but a widespread phenomenon in the animal kingdom (Freedman & Miller, 2008; Güntürkün et al., 2018). Most objects in nature that are behaviorally relevant to animals are variable, even though they might require the same behavioral response (Herrnstein, 1990). Being able to recognize these objects above the individual level and to pool multiple sensory inputs into informative signals with ecological relevance is essential for animals and substantially reduces stimulus complexity (Goldstone & Hendrickson, 2009; Herrnstein, 1990; Jitsumori & Delius, 2001; Repp, 1984). Thus, the organization of the sensory world into perceptual categories is a key component of cognition and facilitates everyday life in a constantly changing environment (Cook & Smith, 2006; Freedman et al., 2002, 2003; Knoblich et al., 2002; Mervis & Rosch, 1981).

Previous studies have employed a variety of different visual stimuli, sometimes with arbitrarily chosen category boundaries that are difficult to control in a systematic manner. To resolve this issue, we have developed a novel stimulus type to study categorization learning: ‘RUBubbles’ are designed as an artificial category stimulus by arranging an arbitrary number of colored spheres in a 3D space. They are generated using custom MATLAB code in which several stimulus parameters can be adjusted and controlled separately, such as the number of spheres, 3D sphere position, size, and color. This approach allows to construct RUBubble categories that are specifically tailored to study various aspects of categorization and category learning.

### Perceptual categorization: Conversion of continuous stimuli into categorical representations

**Fig. 1:**
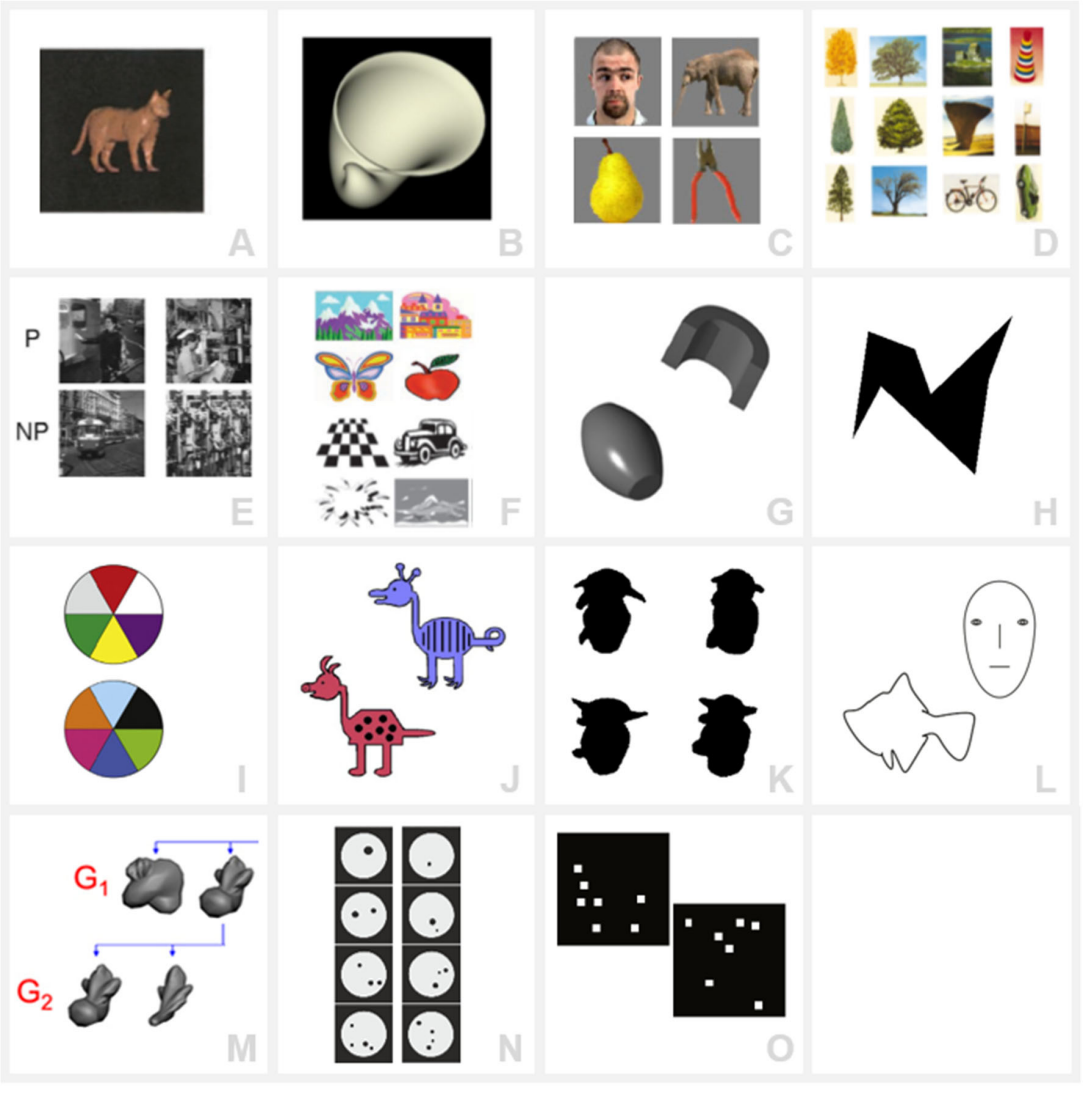
Examples of stimulus sets used in previous studies working on categorization in humans and nonhuman animals. **a** ‘Cat’ morph (From Freedman et al., 2001. Reprinted with permission from AAAS.), **b**shell-shaped object (Reprinted from Acta Psychologica, volume 138, Gaißert, N., Bülthoff, H.H., Wallraven, C., Similarity and categorization: From vision to touch. 219-230, Copyright (2011) with permission from Elsevier via Copyright Clearance Center), **c** animate vs. inanimate (Reprinted from Neuron, volume 60, Kriegeskorte, N., Mur, M., Ruff, D.A., Kiani, R., Bodurka, J., Esteky, H., Tanaka, K., Bandettini, P.A., Matching Categorical Object Representations in Inferior Temporal Cortex of Man and Monkey. 1126-1141, Copyright (2008) with permission from Elsevier via Copyright Clearance Center), **d** tree vs. non-tree (Reprinted with permission from Vogels, R., Categorization of complex visual images by rhesus monkeys. Part 1: behavioural study, and John Wiley and Sons. Copyright © 1999 European Neuroscience Association, European Journal of Neuroscience, 11, 1223–1238), **e** presence or absence of humans (Reprinted from Animal Learning & Behavior, volume 29, Aust, U., Huber, L., The role of item- and category-specific information in the discrimination of people versus nonpeople images by pigeons. 107-119, Copyright (2001) with permission from Psychonomic Society via Copyright Clearance Center), **f**clip-art images (Hampson et al., 2004, Copyright (2004) National Academy of Sciences, U.S.A.), **g** geons (Reprinted from Behavioural Processes, volume 158, Peissig, J.J., Young, M.E., Wasserman, E.A., Biederman, I., Pigeons spontaneously form three-dimensional shape categories. 70-76, Copyright (2019) with permission from Elsevier via Copyright Clearance Center), **h**Attneave-style polygon (produced using the algorithm presented in Collin & McMullen, 2002), **i** color-charts (Reprinted from Behavioural Brain Research, volume 311, Lech, R.K., Güntürkün, O., Suchan, B., An interplay of fusiform gyrus and hippocampus enables prototype- and exemplar-based category learning. 239-246, Copyright (2016) with permission from Elsevier via Copyright Clearance Center), **j** cartoon animals (Copyright (2020) Bowman et al. Created by Bowman, C.R., Iwashita, T., Zeithamova, D., and licensed under CC BY 4.0. Modified. https://elifesciences.org/articles/59360), **k** ‘greebles’ (Reprinted from Vision Research, volume 37, Gauthier, I., Tarr, M., Becoming a “Greeble” Expert: Exploring Mechanisms for Face Recognition. 1673-1682, Copyright (1997) with permission from Elsevier Science Ltd. via Copyright Clearance Center), **l**line-drawings (Reprinted by permission from Springer Nature Customer Service Centre GmbH: Springer Nature, Nature, Visual categorization shapes feature selectivity in the primate temporal cortex, Sigala, N., Logothetis, N.K., Copyright © 2002 Macmillan Magazines Ltd, 2002) **m** digital embryos (Copyright (2012) Journal of Visualized Experiments. Created by Hauffen, K., Bart, E., Brady, M., Kersten, D., Hegdé, J., licensed under CC BY-NC-ND 3.0. https://www.ncbi.nlm.nih.gov/pmc/articles/PMC3598413/, **n** numerosity (Copyright (2020) Ditz and Nieder. Created by Ditz, H.M., Nieder, A., licensed under CC BY 4.0. Modified. https://www.nature.com/articles/s41467-020-14519-2) **o** random dot pattern (Reprinted from Neuron, volume 71, Antzoulatos, E.G., Miller, E.K., Differences between Neural Activity in Prefrontal Cortex and Striatum during Learning of Novel Abstract Categories. 243-249, Copyright (2011) with permission from Elsevier via Copyright Clearance Center)

RUBubbles can be used to generate a continuum between two categories by systematic variation of stimulus parameters. A gradual change of the stimulus parameters is necessary to study one important phenomenon of categorization: the existence of (sharp) category boundaries despite equal physical differences (Freedman et al., 2001). Categorization has been shown to influence sensory perception (Goldstone, 1994; Goldstone & Hendrickson, 2009; Sigala & Logothetis, 2002), and the ability to perceive continuously varying stimuli as belonging to discrete, qualitative categories has been characterized as categorical perception (Repp, 1984). For example, humans tend to group color hues in distinct linguistic categories (Skelton et al., 2017), which might be the reason why we are able to distinguish two colors in certain wavelength ranges but fail in others (Thierry et al., 2009). Perceptual categorization leads to distorted perception such that differences between members of different categories are accentuated whereas differences between members of the same category are attenuated, even when physical differences are actually the same (within-category compression and between category separation)(Goldstone & Hendrickson, 2009; Harnad, 2003). One prominent example of a continuous category stimulus set is the ‘cat and dog’ morphing system (Fig. 1a)(Freedman et al., 2001; Riesenhuber & Poggio, 1999). Freedman et al. used a morphing software to systematically construct morphs between cat and dog prototypes and analyzed categorization behavior and single cell activity in rhesus monkeys. They found clear perceptual category boundaries on the behavioral and neuronal level despite gradual changes in their stimulus set (Freedman et al., 2002; Freedman et al., 2003). Sudden changes in discrimination performance that are not in line with the gradual change of stimulus parameter have also been reported in humans (Emmorey et al., 2003; Harnad, 1987; Liberman et al., 1957), crickets (Wyttenbach et al., 1996), and zebra finches (Caves et al., 2018; Zipple et al., 2019).

### Previous studies on categorization

A large number of studies focused on categorization learning using a wide variety of different stimuli for both human and animal subjects. One common approach is an initial training with several stimuli (discrimination acquisition) and subsequent testing of generalization using novel stimuli to exclude categorization by rote (Güntürkün et al., 2018). In most experimental paradigms, subjects were trained to differentiate between stimuli with a particular object or feature and stimuli without (A vs. not A), or to differentiate between two categories (A vs. B) (Jitsumori & Delius, 2001; Wutz et al., 2018). Performance in transfer tests using unfamiliar stimuli was used as measure of successful open-ended categorization (Herrnstein, 1990). Some exemplary category sets that have been used are animate vs. inanimate (Fabre-Thorpe et al., 1998; Kriegeskorte, Mur, Ruff, et al., 2008b), tree vs. non-tree(Vogels, 1999), Picasso vs. Monet paintings (Anderson et al., 2020; Watanabe et al., 1995), and various clip-art images, pictures, drawings, and photographs (Aust & Huber, 2001; Aust & Huber, 2002; Hampson et al., 2004; Herrnstein & Loveland, 1964; Kreiman et al., 2000) (see Fig. 1c–f for an overview). A key concern with many of those sets is the difficulty to control or identify the stimulus element that was used for categorization (especially with photographs). Category-defining features, for instance the presence or absence of humans (Herrnstein & Loveland, 1964), often co-occur with background features or spatial cues, which might be used by the subjects to categorize the stimuli instead (Aust & Huber, 2001; Güntürkün et al., 2018). Researchers have adopted various strategies to examine which feature their research animals actually relied on for categorization. For instance, the category-defining feature was added to a previously learned negative background and then used in transfer tests (Aust & Huber, 2001). Others used partial masking to cover various stimulus parts and investigated resulting impairments in performance (Gibson et al., 2005) or introduced eye or peck tracking to localize the focus of attention (Dittrich et al., 2010; Freedman et al., 2002).

The use of artificial categories provides a higher control of low-level visual features, which might reduce the need of additional control trials, and facilitates a more direct manipulation of category-defining attributes (Jitsumori & Delius, 2001). Further, artificial stimuli are generally ‘neutral’ without potential confounding effects due to ecological or social relevance unlike stimuli such as faces or food (Vogels, 1999). Previous studies have used basic geometric shapes (geons (Peissig et al., 2019); Attneave style polygons (Attneave & Arnoult, 1956; Collin & McMullen, 2002); rectangle vs. circle (Ashby et al., 1998)), stimuli constructed based on binary, multi-level features (bugs (Smith & Minda, 1998); color-charts(Cook & Smith, 2006; Lech et al., 2016); cartoon animals (Bowman et al., 2020; Bozoki et al., 2006)), nonface objects with common spatial configuration (‘greebles’, (Gauthier et al., 1998; Gauthier & Tarr, 1997)), parameterized line drawings (Sigala & Logothetis, 2002)), digital embryos (created by simulating embryonic development (Hauffen et al., 2012; Hegdé et al., 2008; Kromrey et al., 2010)), abstract numerosity (Ditz & Nieder, 2016), and random dot patterns (Antzoulatos & Miller, 2011; Antzoulatos & Miller, 2014; Wutz et al., 2018) (see Fig. 1g–o).

Our new stimuli offer a way to combine the advantages of such artificial categories with a high degree of customization, while remaining easy to produce with only low costs for computation. By using our RUBubbles, it is possible to precisely control individual stimulus features, and thus to analyze their separate impact on categorization performance. In contrast to already-existing stimulus types, it is feasible to quickly generate a large number of unique RUBubble stimuli, which is crucial to study categorization learning. By combining specific generation algorithms and training paradigms, different characteristics and strategies of categorization learning can be investigated on a behavioral and neuronal level. The following sections will explain in detail, how RUBubble stimuli and specific category sets are generated and how each stimulus parameter can be manipulated individually.

## Creation of RUBubble stimuli

We provide ‘RUBubble stimuli’ as an easy-to-useMATLAB-based application. RUBubble stimuli can be controlled purely programmatically or via a graphical user interface (‘*RUBubblesAPP*’). All MATLAB files are freely available and can be customized or expanded depending on individual needs (code can be downloaded from https://gitlab.ruhr-uni-bochum.de/ikn/rububbles, published under the terms of the Creative Commons Attribution License, which permits unrestricted use and redistribution provided that this article is cited to credit the original authors). An additional MATLAB live script illustrates the creation process and ensure a user-friendly testing and implementation. The RUBubblesAPP can be freely downloaded as well and used as MATLAB app or independently as standalone desktop app without a MATLAB license. For a full list of features, user manual, etc. we have setup a Wiki (https://gitlab.ruhr-uni-bochum.de/ikn/rububbles/-/wikis/home).

**Fig. 2:**
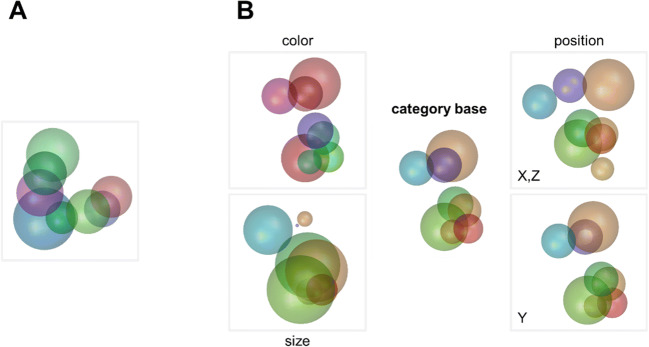
‘RUBubbles’ as novel stimulus type to study categorization. **a** RUBubble stimulus as an arbitrary number of colored spheres in 3D space. **b** Generation of novel stimuli based on a category base. Depending on the parameter specifications, distinct features of RUBubble stimuli vary to different degrees. For example, a new stimulus can be created to have a similar position and size of the spheres but be highly variable in color (*upper left stimulus*). Alternatively, a stimulus could show similar color and size but very different sphere positions (*upper right stimulus*)

RUBubble stimuli are composed of an arbitrary number of colored spheres that are arranged in 3D space (Fig. 2). Three parameters control the stimulus appearance and can be adjusted and manipulated separately: the color, position, and size of each sphere (Fig. 2b). We have developed several MATLAB functions, which are also implemented in a graphical user interface (‘*RUBubblesAPP*’), to generate various stimulus sets for categorization learning. The first step in stimulus generation is to define a base stimulus for each category. Subsequently, all other category members are generated as derivatives of the base stimulus. Three different main calculation methods can be used to produce a RUBubble category and will be explained in the following sections (for an overview, see Table 1 and the overview table within the Wiki documentation).

**Table 1:**
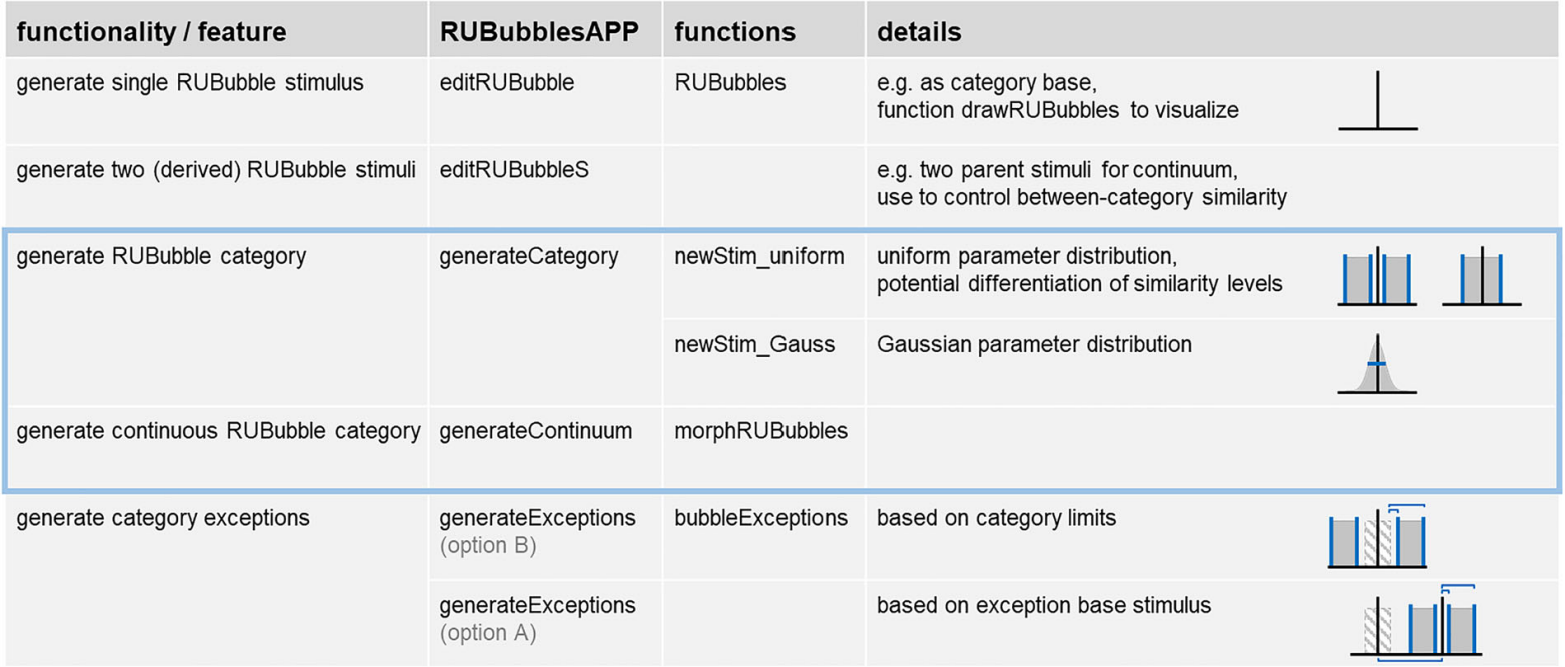
Overview of MATLAB functions and specific RUBubblesAPP components to create RUBubble stimuli for categorization experiments. The three main calculation methods are highlighted

Our specifically designed RUBubblesAPP allows an easy, user-friendly manipulation of a wide variety of additional stimulus features. Besides the high level of customization this APP also facilitates the use and testing of RUBubble stimuli away from the code-based implementation and outside of the MATLAB environment.

### Category base stimulus

**Fig. 3:**
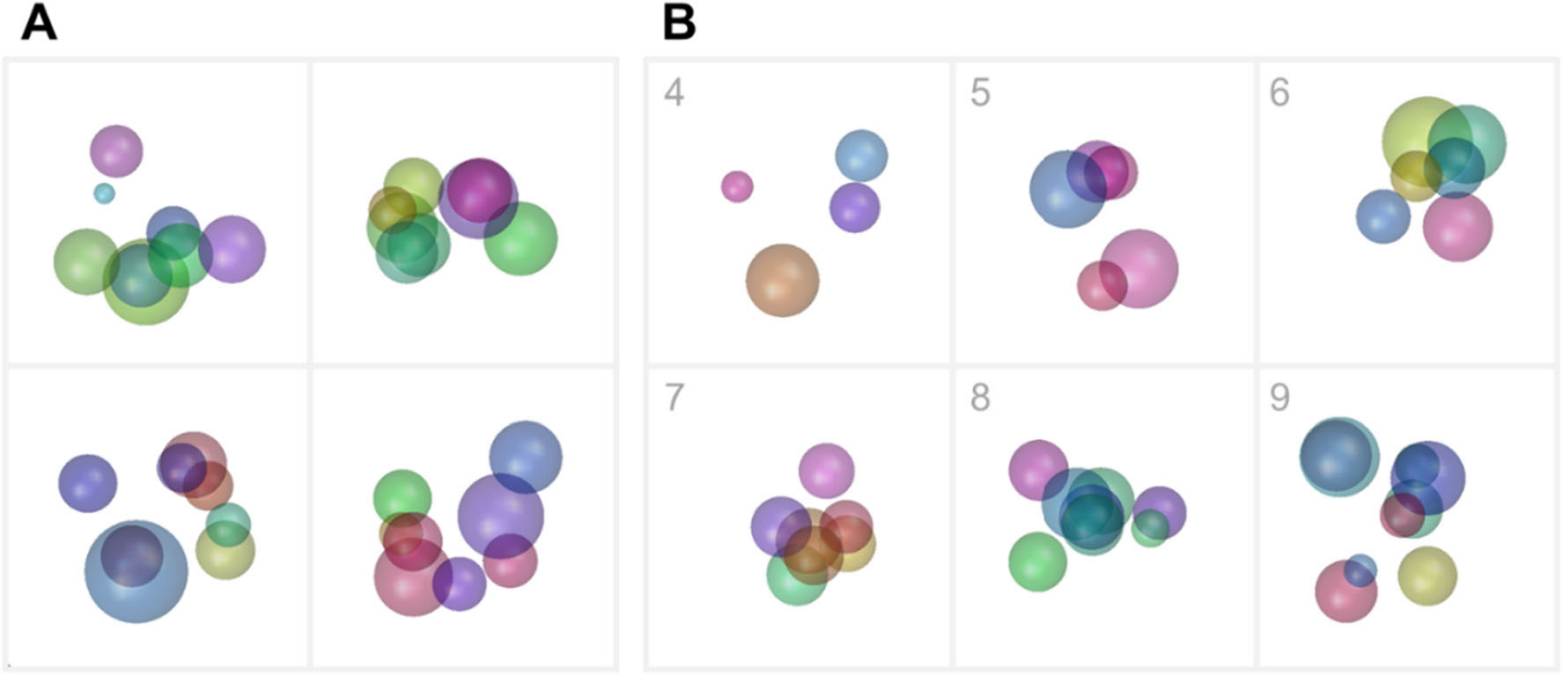
Examples of randomly generated RUBubble stimuli using the function ‘*RUBubbles*’. The input argument of this function determines the number of spheres. **a** RUBubble stimuli consisting of eight spheres, generated by separate calls of ‘*RUBubbles(8)*’. **b** RUBubble stimuli consisting of 4–9 spheres, each generated by calling ‘*RUBubbles*’ with the respective number of spheres as input argument, e.g., ‘*RUBubbles(5)*’ (sphere number indicated in upper left corner of each stimulus). An additional function that is necessary to visualize RUBubble stimuli is explained below

**Fig. 4:**
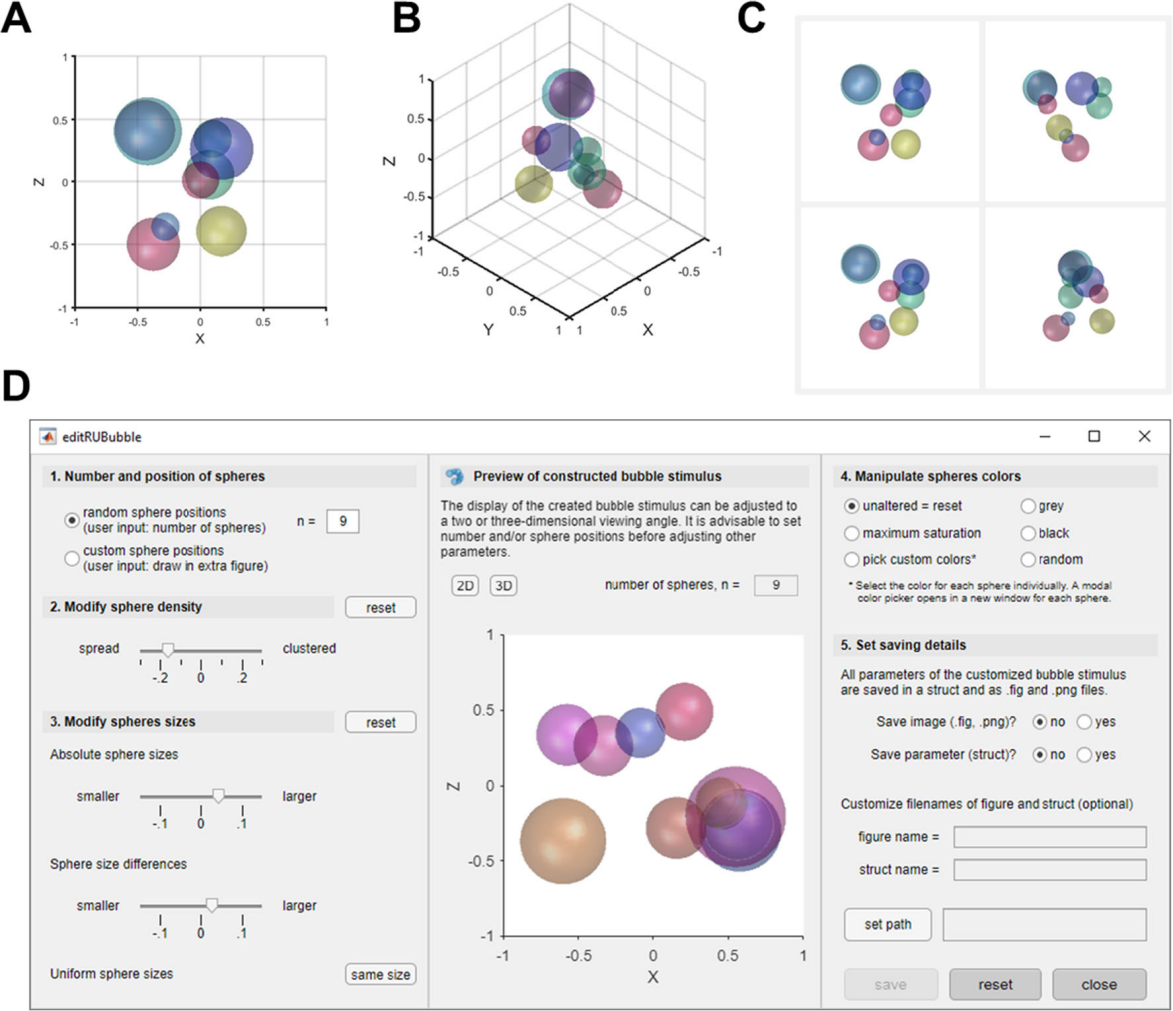
Display of stimulus axes, different viewing angles and stimulus preview within the RUBubblesAPP. **a** Default, two-dimensional view of a RUBubble stimulus along the *x*-*z* axes. **b**Three-dimensional view of the same stimulus along the *z*-*y*-*x* axes. **c** Various, manually adjusted viewing angles of the stimulus shown in A and B with axes turned off. RUBubble stimuli are visualized using the function ‘*drawRUBubbles*’. **d** Screenshot of the app component ‘*editRUBubble*’, which can be used for targeted stimulus generation. The preview in the center column directly visualizes all post hoc stimulus customization

A category base stimulus can be generated using the function ‘*RUBubbles*’ or via the ‘*editRUBubble*’ component within the app. In both cases, the desired number of spheres is the only mandatory input argument (Fig. 3). All other stimulus parameters (*X*, *Y*, *Z* coordinates, size, and color) are randomly generated, but can later be customized when using the app (e.g., modification of sphere density, coloring and size, Fig. 4d). In the app, the user can further mark 2D sphere positions and thereby define the general spatial arrangement of all spheres instead of specifying the sphere number.

#### Visualization of RUBubble stimuli

RUBubble stimuli generated in MATLAB can be visualized using the function ‘*drawRUBubbles*’, which requires all previously generated stimulus parameters as input argument (see live script ‘bubbles.mlx’). The output is a MATLAB figure that contains the RUBubble stimulus as a 3D object, which is shown along hidden axes (default mode, Fig. 3). It is possible to show the axes for clarification (Fig. 4a, b) or to modify the viewing angle and rotate the stimulus to, for instance, generate 2D images of the same stimulus at different viewing angles (Fig. 4c). RUBubble stimuli can be saved in various image formats (such as .jpg, .png, and MATLAB .fig) and exploited as basis for 3D objects to be used in, for instance, human fMRI studies.

Within the app, a preview of the created RUBubble stimulus is continuously updated to immediately visualize customizations (Fig. 4d). All stimulus parameters and a figure of the generated stimulus can be saved to local folders and used at a later stage for the generation of a full category.

## Generation of RUBubble categories

**Fig. 5:**
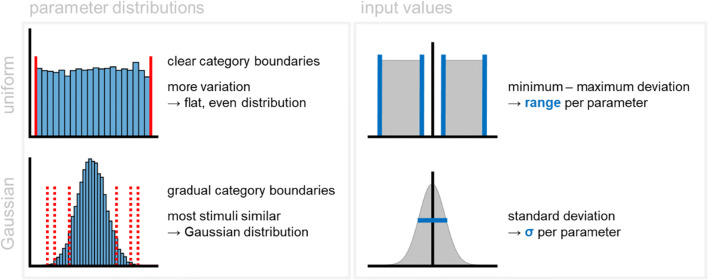
Characteristics of RUBubble categories resulting from different generation methods and input types. A category calculated based on minimum–maximum deviation ranges per parameter comprises more variation since all features are equally likely and clear boundaries exist (uniform distribution). The calculation of a category based on the standard deviation per parameter results in mostly similar stimuli but vague category boundaries (Gaussian distribution)

All members of a RUBubble category are generated as derivatives of a category base. Parameters of novel stimuli are pseudo randomly produced using either a Gaussian, or a uniform distribution. Thus, novel stimuli are created by defining either the range (δ), or the standard deviation (σ) of each stimulus parameter relative to the category base. The different calculation methods are implemented in two different functions, which require all parameters of the category base stimulus (mandatory), and a range or standard deviation (optional) to control the deviation of the calculated values from the base stimulus (Fig. 5). The number of spheres remains the same for all stimuli within one category in both methods.

Depending on the parameter distribution used, RUBubble categories differ in their feature distribution (Fig. 5). Using uniformly distributed pseudorandom numbers within a specific range results in clear category boundaries and more or less equally frequent stimulus parameters. Thus, stimuli with higher dissimilarity to the category base are as frequent as stimuli with minor variation. A stimulus set generated based on a Gaussian distribution contains few strongly divergent stimuli, while most category members exhibit similar features. As a result, a clearly defined category border is missing.

When working in MATLAB, the user can choose the desired underlying parameter distribution by selecting the corresponding function (‘*newStim_uniform*’ or ‘*newStim_Gauss*’, see live script ‘bubbles.mlx’ for an example). A range that specifies the minimum and maximum deviation of each parameter is used as additional input argument for the function ‘*newStim_uniform*’. The values that are added or subtracted from the value of the base stimulus are drawn from the given range (marked in grey, see upper right schema in Fig. 5).

Alternatively, novel stimulus parameters can be selected from a Gaussian distribution with the value of the category base as mean (category base delineated by the vertical black line, parameter distribution of novel stimuli outlined by grey shaded Gaussian distribution, Fig. 5). The width of the distribution is defined by the standard deviation σ, which is used as an additional input argument for the function ‘*newStim_gauss*’. Sigma defines the amount of variation, and the extent to which parameters of novel stimuli vary from the category base. A low standard deviation indicates that stimulus parameters tend to be close to the base stimulus, whereas a high standard deviation indicates a wider spread. Thus, the higher the standard deviation, the higher the percentage of stimuli with larger deviations from the category base.

**Fig. 6:**
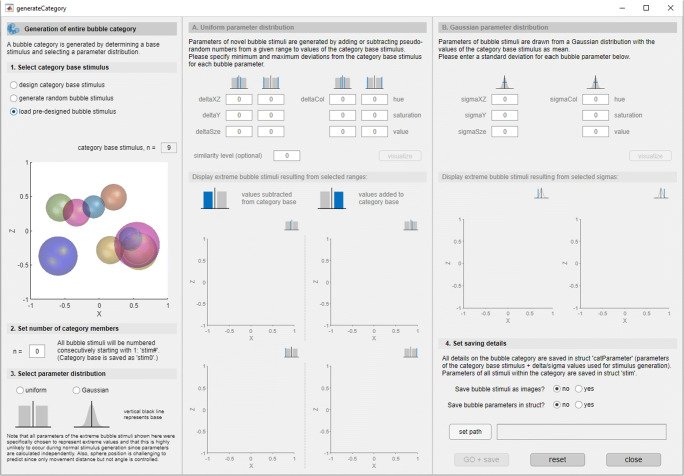
‘*generateCategory*’ component of RUBubblesAPP to create a full RUBubble category. After a category base was picked (*left column*), the user can select one of the two parameter distributions, which then enables the respective input fields in the middle (uniform distribution) or right (Gaussian distribution) column. Figures in the respective column visualize examples of RUBubble stimuli created based on the extreme values (*middle*) or at 5% from the tails of the Gaussian distribution (*left*)

Using the RUBubblesAPP, the user simply selects the desired parameter distribution after having specified a category base stimulus (‘*generateCategory*’ component within the app, Fig. 6). This, in turn, enables entering of either minimum and maximum deviation values or standard deviations per stimulus parameter.

The calculations per stimulus parameter for both methods are briefly described in the following paragraphs and explained in more detail in the Wiki documentation. Aside from different parameter distributions, there are other category features that can be controlled when creating RUBubble categories, such as within- and between-category similarity, or the number of category prototypes (see Wiki for more information).

### Sphere size

**Fig. 7:**
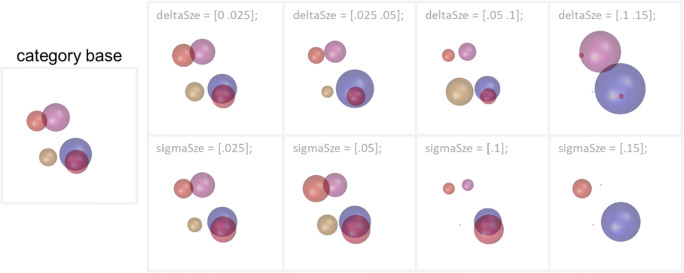
Small numerical changes of the sphere size parameter can result in significant changes of the visual display. Only the size parameter was altered during the generation of several RUBubble stimuli to illustrate the effect of various ranges and standard deviations on the resulting sphere sizes. The delta and sigma values that were used for the parameter generation are indicated above each stimulus. Undersized spheres become more likely for larger deviation ranges and standard deviations (rightmost stimuli, earlier using Gaussian distributions for stimulus generation). Spheres with sizes below 0.01 become barely visible and are thus set to 0.01 as minimum size value

Sphere sizes are either calculated by adding or subtracting uniformly distributed pseudorandom numbers to values of the category base stimulus or selected from a Gaussian distribution with the value of the category base as mean (Wiki, Figs. 1 and 4). Relatively small numerical changes in size values result in a noticeable visual change of sphere size. Figure 7 provides an overview of RUBubble stimuli that were generated using different ranges or standard deviations (all other stimulus parameters unaltered and thus identical to the category base stimulus). As illustrated in Fig. 7, the use of a small range closer to the category base stimulus or a low standard deviation is advisable.

### Sphere color

**Fig. 8:**
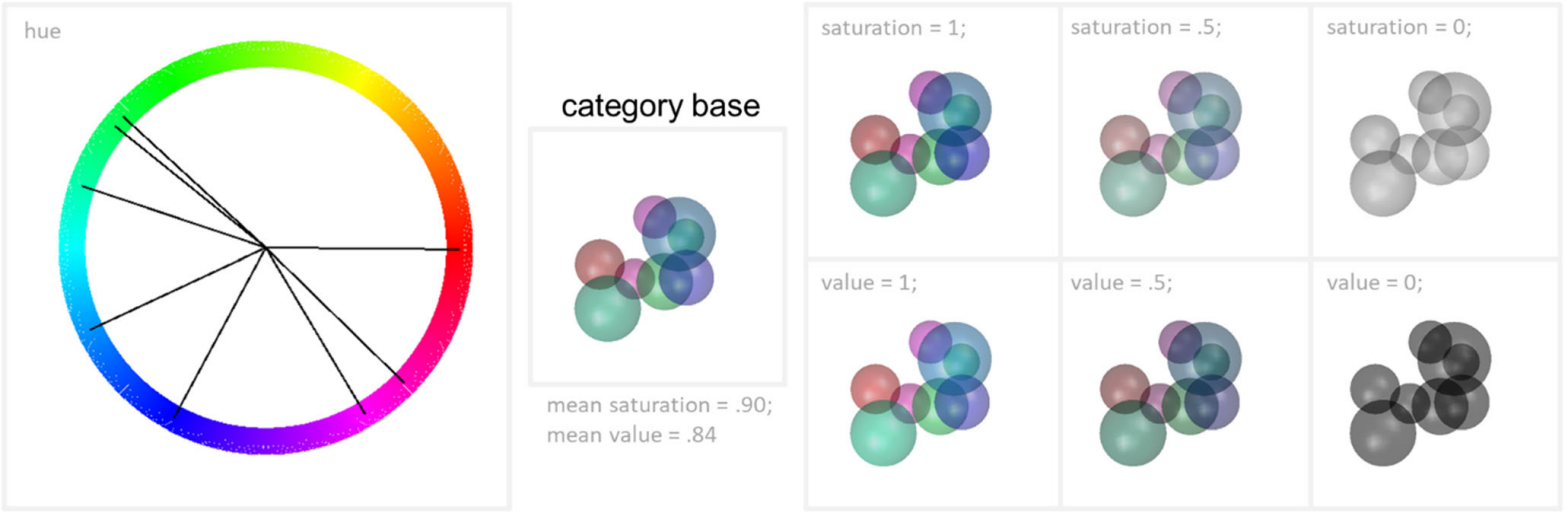
Specification of sphere colors. *Hues* of all spheres of the depicted category base stimulus are indicated via the *black lines* in the circular color space. The effects of distinct values of saturation (amount of color, *upper row*) and value (relative brightness, *bottom row*) are shown by selective alteration of the respective color parameter as indicated above each image. The initial values for saturation and value of the category base stimulus were .9 (saturation) and .84 (value). Colors become increasingly pale with a decrease of saturation and progressively darker with decreasing value

Color values are calculated in the same way as sphere sizes (Wiki, Figs. 1 and 4). The calculations are performed within the HSV color space; thus, RUBubble colors are described by their hue (shade of color), saturation (amount of color), and value (relative brightness). Hue is described on a chromatic circle with red being defined as both 0 and 1 (Fig. 8). Saturation and value describe the amount of color and relative brightness. Color parameter can be modified individually although they are perceptually linked. To prevent the occurrence of colorless, pale, or simply black spheres it is advisable to use values resulting in minor deviations when manipulating saturation and value or to leave both color parameters unaltered (Fig. 8).

### Sphere position

**Fig. 9:**
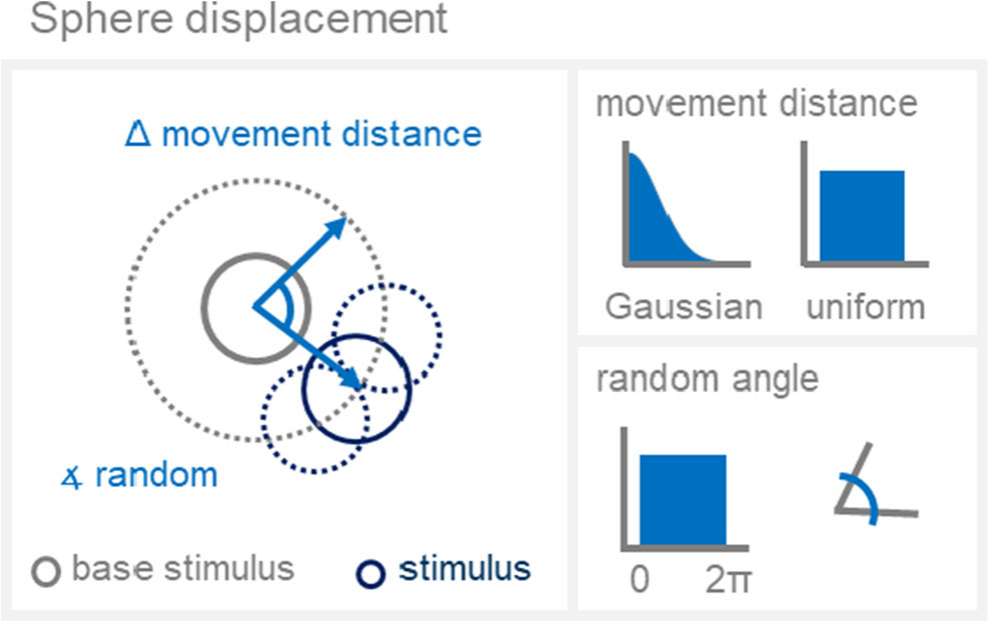
Schematic description of the calculation of novel sphere positions. *Spheres* are shifted in polar coordinates based on movement distance (specified by user input) and angle (random, uniformly distributed within 0 and 2π). Generated polar coordinates are transformed back into Cartesian coordinates, which are then added (or subtracted) to the coordinates of the category base stimulus

To alter their position, all spheres are shifted by a given distance, in a random direction using a polar coordinate system (Wiki, Figs. 3 and 6). Angular values are drawn from a uniform distribution bounded by 0 and 2π and the distances are selected from either a uniform distribution within a given range or a Gaussian distribution (Fig. 9).

In our experiments, RUBubble stimuli were displayed along the *x*-*z* axes as 2D images. Thus, the *Y* coordinate of each new stimulus only affected the order of spheres and co-varied with changes in sphere size. To reduce the degrees of freedom for position changes and to retain a higher similarity between RUBubble stimuli, the *Y* coordinate is calculated independently from the *X*-*Z* coordinates in the same way as size and color parameters. Yet, the MATLAB code could be customized such that sphere positions change on all three axes when generating novel stimuli in 3D.

## Generation of a continuous category

**Fig. 10:**
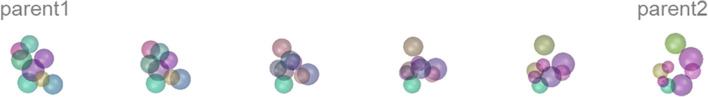
Example of a continuum between two RUBubble stimuli. Parent1 is gradually morphed into parent2, both of which must consist of the same number of spheres. Which pair of spheres will be morphed into each other is unsupervised and random (follows the order of spheres in both input structs)

A continuum between two RUBubble stimuli is generated using the function ‘*morphRUBubbles*’ or the RUBubblesAPP component ‘*generateContinuum*’ (Fig. 10). The number of in-between stimuli and the parameters of two RUBubble stimuli with an equal number of spheres are required as input arguments. Morphing is based on stimulus parameters instead of visual features, which also explains why an equal number of spheres in both parent stimuli is necessary (i.e., sphere 1 of parent1 will be morphed into sphere 1 of parent2). So far, the mapping of individual spheres that are morphed into each other is unsupervised and random. To morph according to sphere position, color, or size would require an extension of the existing code, just as morphing of RUBubble stimuli with differential numbers of spheres. An example on how to gradually morph two RUBubble stimuli is included in the MATLAB live script (‘bubbles.mlx’).

## Generation of category exceptions

**Fig. 11:**
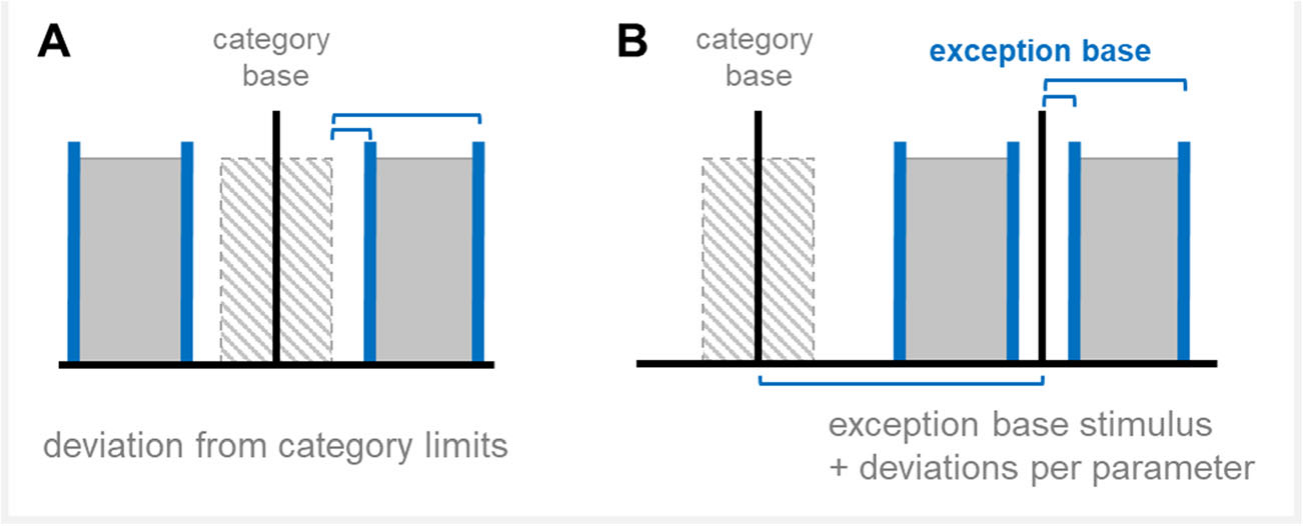
Schematic illustration of two possible approaches to generate category exception stimuli. **a** Category exceptions can be generated by defining the minimum and maximum deviations from the borders of a specific input category (MATLAB function ‘*bubbleExceptions*’, option B in RUBubblesAPP ‘*generateExceptions*’). **b** Alternatively, category exceptions can be created by designing an exception base stimulus and using min/max ranges per parameter (option A in RUBubblesAPP ‘*generateExceptions*’). The latter approach enables different sphere numbers between category and exception stimuli and the definition of exact differences between category base and exception base stimulus

Category exceptions are stimuli that do not belong to a given category on a perceptual level. They can be generated as independent stimuli and then be assigned to a category. However, in order to control how much and in which respect they differ from all other members of a category, exceptions need to be constructed in a specific way. The function ‘*bubbleExceptions*’ generates RUBubble stimuli that exhibit specific deviations from an input category but have a consistent number of spheres (Fig. 11a).

The stimulus parameters of category exceptions are calculated based on deviations from the outmost or extreme values of a category. Thus, the upper and lower parameter limits of each RUBubble parameter have to be calculated first (see Wiki). Stimulus parameters of category exceptions are then calculated by either adding values to the upper limit of the category or subtracting values from the lower limit (Fig. 11a). The MATLAB live script gives an example on how to generate category exceptions for a previously generated RUBubble category (‘bubbles.mlx’). Note that even if minimum deviation values are set to 0, the resulting parameter values represent the extreme values of the category and can thus differ profoundly from the category base stimulus or other category members.

The RUBubblesAPP component ‘*generateExceptions*’ allows another, additional approach to generate exceptions besides the previously described procedure. Here, the user specifically designs a base stimulus for all category exceptions (exception base stimulus, Fig. 11b). Similar to the generation of a RUBubble category, a min/max range per stimulus parameter then defines the potential deviations from this exception base stimulus.

## Categorization learning training protocols

One major influencing factor on different categorization strategies is the way in which categories are experienced, or, in experimental studies, the behavioral training protocol. Whether the category is initially learned with only few, highly representative stimuli or a larger set of multiple exemplars should affect how it is represented. Categories can differ in the underlying structure and the distribution of characteristic features. For instance, they can be defined based on a common prototype or on similar features present in multiple exemplars. These different category structures are thought to encourage different strategies, which also involve distinct brain areas. With RUBubble stimuli, it is possible to generate different category structures that can be used to investigate differences in categorization learning. We propose three different training protocols that can be used with distinct RUBubble categories to study and compare different aspects of categorization learning.

### Prototype- and exemplar-based categorization

It was initially shown for color that not all category members are equally representative of their category (‘focal colors’ as best examples similar across languages (Heider, 1972; Mervis & Rosch, 1981)). In general, most representative category members are usually learned first, show high within-category similarity, and simultaneously share the fewest attributes with contrasting categories (Mervis & Rosch, 1981). An initial training with only representative stimuli was shown to be more accurate, faster, and ‘superior to training on a range of examples’ (cf. Mervis & Rosch, 1981, Mervis & Pani, 1980). The most typical, ideal example or central tendency of a category is usually referred to as category prototype (based on means, or ideal values, (Goldstone & Hendrickson, 2009); ‘schema’ (Posner & Keele, 1968); ‘super stimulus’ (Jitsumori & Delius, 2001)). The similarity to this prototype, which combines all category-defining features, defines whether a stimulus belongs to the same category or not. Thus, an abstract prototype facilitates categorization of a large number of stimuli since it allows generalization (Jitsumori & Delius, 2001). In contrast, exemplar-based categorization strategies require an extraction of common elements or central features from several category exemplars. Each novel category stimulus is compared to all exemplars in both categories and then assigned to the category it shares the most features with (Minda & Smith, 2001). Category-defining features must be identified and derived from multiple exemplars, which requires a certain familiarity with several exemplars and explains slower initial learning (Minda & Smith, 2001). This abstract and conceptual collection of category-defining features can later be used to determine category membership comparable to a category prototype (‘formation of a prototype […] [as] means of classification’ (Reed, 1972)). Several characteristics of a category, such as category size, structure, and stimulus complexity, promote different strategies in category learning. According to Minda and Smith (2001) initial category learning is based on prototype-based strategies, whereas exemplar-based strategies emerge as secondary processes along with correct categorization of exceptions, which cannot be explained solely on prototype-based categorization (Minda & Smith, 2001). Small set sizes favor exemplar-based strategies such as memorization, whereas large numbers of complex stimuli promote prototype-based strategies (Minda & Smith, 2001). Besides differences in categorization behavior, the underlying neuronal dynamics and involved brain regions have been shown to differ depending on the respective categorization strategy. For instance, prototype representations were found in the (ventromedial) prefrontal cortex, (anterior) hippocampus, and medial temporal lobe (Bowman et al., 2020; Lech et al., 2016). In contrast, exemplar representations were demonstrated in the inferior frontal gyrus and lateral parietal cortex (Bowman et al., 2020). The behavioral training protocol used in experimental studies further affects which categorization strategy might be favorable.

### Prototype-based

**Fig. 12:**
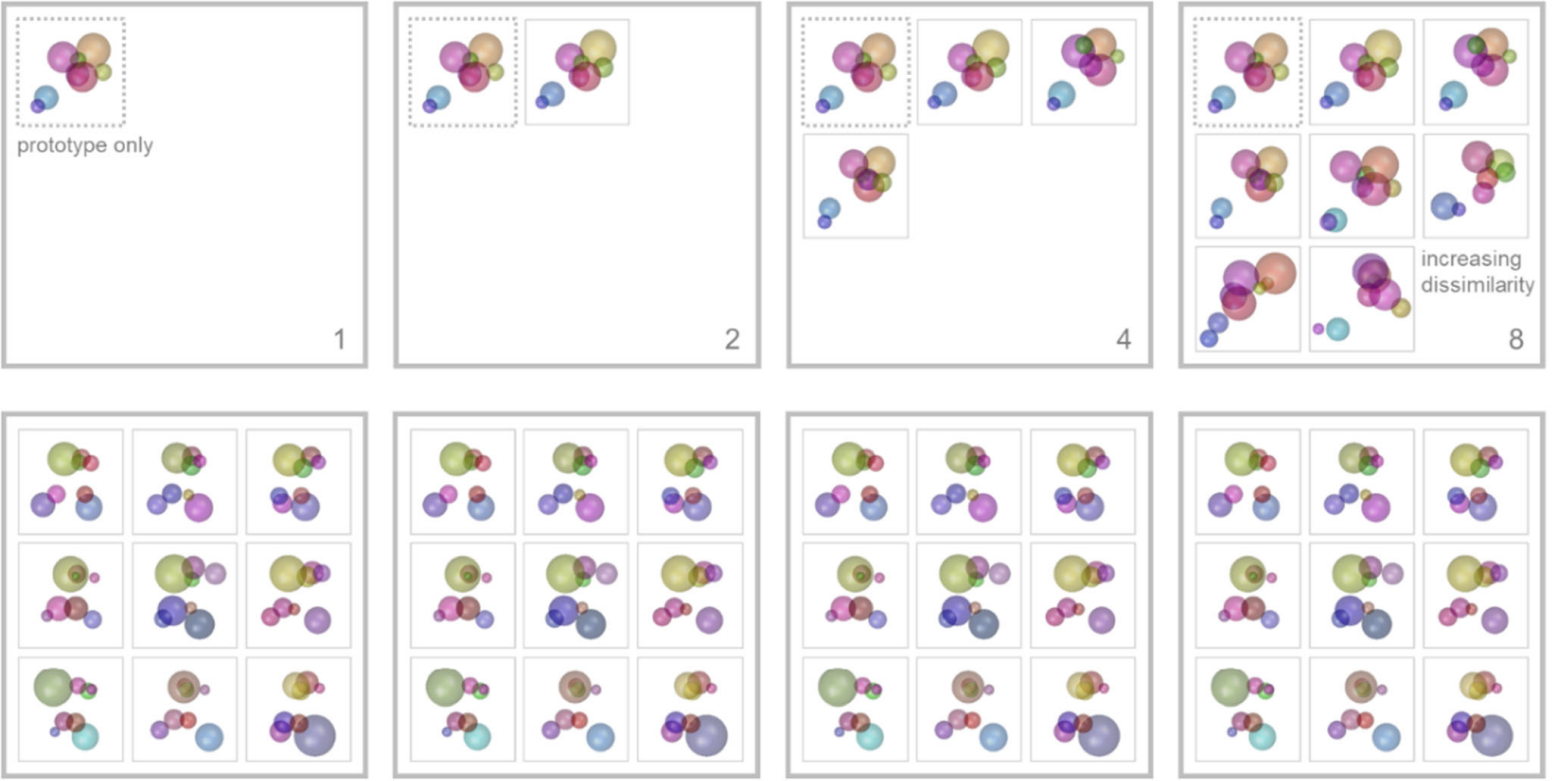
Prototype- and exemplar-based training protocols differ mainly in the selection of stimuli at the beginning of each session. Whereas in prototype-based protocols stimulus number is gradually increased (initially only prototypes, performance-dependent doubling of stimulus number, top), exemplar-based protocols draw stimuli ab initio from the entire stimulus pool (no subdivision in training blocks with distinct number of stimuli, category base stimulus not included in stimulus set, bottom)

In a prototype-based training protocol, we suggest to successively increase the number of stimuli depending on the behavioral performance. The subjects initially perform the task with both category prototypes only, which exhibit all category-defining characteristics and central features as category base stimuli (delayed match to sample, Fig. 12). Once they perform at or above a certain behavioral criterion (e.g. 80 % correct) the stimulus number is increased. Thus, the task changes from a delayed match to sample to a delayed match to category paradigm, due to an increasing number of category stimuli. Each session can be subdivided into a set of training blocks, each of which contains a specific number of stimuli per category (compare with the prototype distortion paradigm used by Antzoulatos & Miller, 2011, see example 1, Wiki GitLab). Consequently, the final stimulus number per session is dependent on the performance. Along with an increasing set size, the percentage of novel stimuli also increases (the larger the stimulus set used, the less likely that a randomly selected stimulus was already familiar), which further enhances task difficulty. Due to the small number of highly representative stimuli at the beginning of each session (i.e. only prototypes), the learning curve should be steep at the beginning and then gradually flatten throughout the training session (higher percentage of unfamiliar stimuli as a result of performance-dependent increase in stimulus number). This should be more pronounced for stimulus sets that are composed of stimuli with gradually decreasing similarity to the prototype (i.e. the higher the stimulus number the more dissimilar). RUBubbles present an ideal stimulus type to use this training approach, since large numbers of unique stimuli can be generated.

### Exemplar-based

An exemplar-based training protocol differs in the way stimuli are selected. Instead of restricting the stimulus set early in the session to few, highly representative stimuli, all stimuli are randomly chosen from the entire stimulus pool (Fig. 12). Hence, the likelihood to encounter unfamiliar stimuli early in training is very high. This training approach is more difficult due to the unrestricted stimulus set and the fact that category features have to be extracted from various exemplars and cannot be directly deduced from a prototype. Therefore, the learning curve should exhibit a slower initial increase and overall flatter slope in comparison with a prototype-based training session. A flattening of the curve towards the end of the session should, in contrast, be less pronounced.

### Continuous

In a training protocol using a continuum between two RUBubble stimuli, subjects have to assign individual sample stimuli to one of the two categories. Variations between adjacent stimuli are gradual, thus, it is possible to investigate whether subjects experience a sharp category boundary despite identical physical differences between stimuli. It is also possible to train subjects on arbitrary category boundaries using a continuous stimulus set and compare changes in neuronal activity when these boundaries are modified and have to be relearned by the subjects (compare with (Freedman et al., 2002)).

## Discussion

We introduced RUBubbles as a novel stimulus type to study categorization learning. RUBubbles show several desirable characteristics and represent a valuable supplement for categorization research, which adds to a large number of diverse stimulus types that have already been used in this field (see Fig. 1 for a small selection). RUBubbles can be used to quickly generate large stimulus sets, allow for specific manipulation of individual stimulus parameters, and enable users to adjust the existing code to suit individual requirements. The degree of variability within and between categories can be manipulated via precise specification of stimulus parameters, and a continuum can be produced using two starting RUBubble stimuli.

As an example, the spatial arrangement of individual spheres can be deliberately manipulated by defining the desired movement distance when generating novel stimuli. Hence, it would be possible to study the sensitivity to configural changes as is done in, for instance, face perception research (Gauthier & Tarr, 1997). ‘Greebles’, artificial nonface object stimuli, have been used to disentangle face-specific sensitivity from more general mechanisms, such as specialized knowledge or expertise with visually similar objects (Fig. 1k, Gauthier & Tarr, 1997; Gauthier et al., 1998). These stimuli are three-dimensional objects that share similar elements in common spatial configurations and can be categorized on different levels. RUBubbles can also be generated to represent multiple levels for categorization (for instance by using categories with varying sphere numbers as basic level). The straightforward generation of RUBubble stimuli might facilitate studies that require larger sets of unique stimuli and thus RUBubbles might provide a useful addition to object recognition research.

Nonetheless, some aspects remain that still need to be considered and our RUBubble framework is intended as a live product providing a strong fundament that can be further developed for which we are happy to receive any extension or improvement suggestions.

**Fig. 13:**
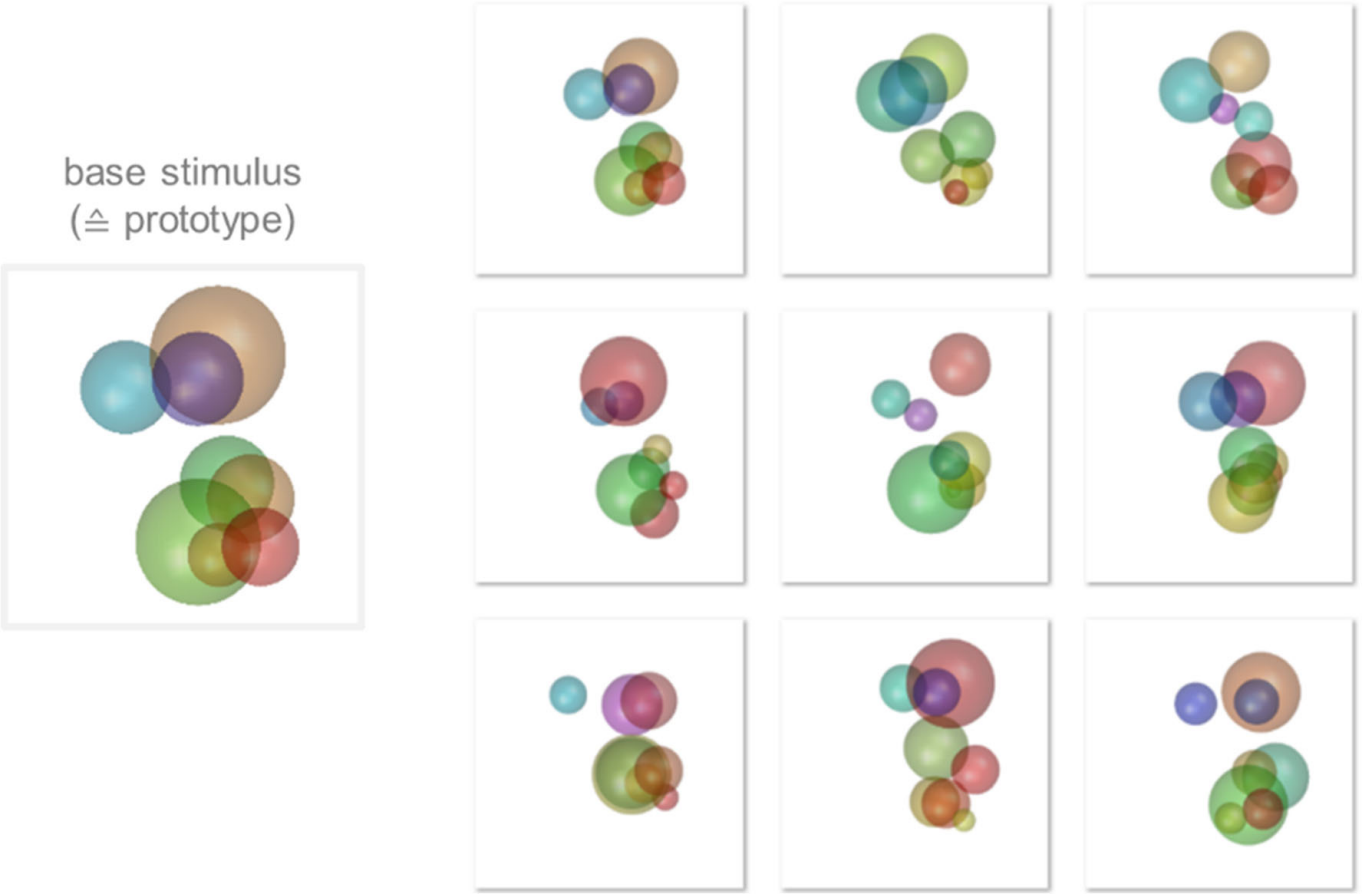
When viewing RUBubble stimuli, several questions come to mind regarding the similarity between, for instance, the category base stimulus as prototype and all other category members. Such as, do spheres form cluster? How far are spheres generally spread? Does this change for other category members compared to the prototype? Are spheres moved further away or closer together? How much do sphere colors vary within the prototype and are they more or less variable in each category member?

Naturally, perceptual similarity is challenging to determine for any visual stimulus. This applies to RUBubbles as well as any other already existing category stimulus type. RUBubble stimuli can be subdivided into distinct similarity levels based on mathematical properties, i.e. the deviation of parameter values from the category base stimulus (within-category similarity) or from the base stimulus of a second category (between category similarity). After generating a RUBubble category, the Euclidean distance of each sphere relative to the category base stimulus, and the absolute deviation in size and color values can be calculated for each stimulus (basic, mathematical similarity that provides indications of within-category similarity). However, it remains extremely difficult to interpret such deviations with regard to perceptual similarity, since large changes in numerical parameter values might lead to hardly visible changes in the visualized stimulus, whereas minor changes might completely alter the overall stimulus appearance. When looking at RUBubble stimuli it is obvious that the general impression most likely is more significant than numerical deviations of stimulus parameter (Fig. 13).

The different characteristics of RUBubble stimuli (i.e., color, size, spatial arrangement/position) most likely differ in salience depending on the specific stimulus set, training procedure, or subject. It is plausible that different participants or research animals use different stimulus features for categorization or that they adjust their focus depending on the respective stimulus set properties. Categorization training has been shown to influence perceptual sensitivities and to affect the salience of specific stimulus features (via acquired distinctiveness or acquired equivalence) (Goldstone, 1994). For instance, after having learned to categorize RUBubble stimuli mostly based on differences in sphere size, subjects most likely initially focus their attention on this specific stimulus feature when learning a new set. Besides, despite additional adjustment trials to produce equally discriminable stimuli, Goldstone (1994) reported a clear difference in processing of different dimensions (Goldstone, 1994). A more practical approach to identify the most prominent category-defining feature might be to evaluate which feature subjects relied on most after having learned a specific RUBubble set (e.g., by asking participants or calculating a GLM using performance data and RUBubble stimulus properties). RUBubble categories can be generated to encourage subjects to using specific parameters for categorization (by adjusting the respective variation per parameter within a category or manipulating the between category similarity). Ultimately, controlling and manipulating the saliency of different stimulus dimensions remains the responsibility of the individual researcher and has to be fitted to the respective experimental design and research question.

Alternative methods to assess the perceptual similarity that have been implemented in previous studies to evaluate the similarity of 2D images are multidimensional scaling, MDS (Hegdé et al., 2008), and representational similarity analysis, RSA (Kriegeskorte, Mur, & Bandettini, 2008a). Both could be applied to RUBubble stimuli but are complex, time-consuming, or lack the color dimension. In general, it is difficult to obtain any similarity value without making (too many) assumptions on how the subjects actually perceive the stimuli or on which stimulus parameter they rely for categorization (see Caves et al., 2019 on human biases when studying animal perception).

For instance, color vision differs in various degrees for different animals or even individuals in one species, especially birds show pronounced differences to mammals, which results in quite different color perception. Most monitors are calibrated for human subjects and thus might need other color adjustments when used with nonhuman animals. RUBubble colors are defined in the HSV color space and although all three color parameters (hue, saturation, and value) can be manipulated independently, these parameters represent integral dimensions that are not perceptually separable (Burns and Shepp, 1988; Gottwald and Garner, 1975). Thus, color is generally perceived holistically and classified based on overall similarity rather than on separable dimensions (Goldstone, 1994), which should be kept in mind when manipulating RUBubble color. Besides, RUBubble stimuli can easily be transformed into various shades of grey or black instead or defined within another color space by adapting the code. Another possibility would be to keep sphere colors within one category consistent. In general, a more suitable approach to determine similarity might be to analyze how strongly each stimulus parameter affects the behavioral performance and then use this knowledge to more precisely construct the following RUBubble categories.

Nonetheless, the here presented methods for RUBubble generation allow the creation of countless unique stimulus sets. Typically, stimulus sets contain two RUBubble categories, but they can also be designed to comprise multiple categories instead. The MATLAB code and the complementary RUBubblesAPP are easy to use, freely available, and do not require any additional toolboxes. Using the app allows to generate custom stimulus sets without prior programming experience or a MATLAB license. Besides, RUBubbles are immensely versatile and can be used to study diverse questions in categorization research, such as categorical perception of a sensory continuum or the effect of stimulus statistics on categorization learning.
